# Laparoscopic Heller–Dor as salvage therapy after failed POEM: a prospective comparative study

**DOI:** 10.1007/s00464-026-13278-3

**Published:** 2026-08-19

**Authors:** Irene Sole Zuin, Tommaso Garbo, Luca Provenzano, Francesca Forattini, Giovanni Capovilla, Matteo Santangelo, Arianna Vittori, Matteo Pittacolo, Giulia Nezi, Matteo Ghisa, Edoardo Vincenzo Savarino, Lucia Moletta, Loredana Nicoletti, Michele Valmasoni, Renato Salvador

**Affiliations:** Department of Surgical, Oncological and Gastroenterological Sciences, School of Medicine, University of Padua, Padua, Italy

**Keywords:** Esophageal achalasia, Heller myotomy, Pyloromyotomy, Treatment outcome

## Abstract

**Objectives:**

Current therapeutic options for esophageal achalasia (EA) include pneumatic dilation (PD), laparoscopic Heller–Dor myotomy (LHD), and peroral endoscopic myotomy (POEM), which has gained increasing popularity over the last decade. However, symptoms persist or recur after POEM in 10–20% of cases. Evidence on management of POEM failures is limited to small case series. This study evaluates the outcome of LHD in patients who previously underwent endoscopic treatment—POEM or PD—as compared to treatment-naïve patients.

**Methods:**

Consecutive patients with EA who underwent LHD between 2020 and 2025 were prospectively recorded. The study population was divided in three groups: treatment-naïve (NA group), prior POEM (POEM group), and prior PD (PD group). Primary outcomes were morbidity, mortality, and therapeutic success. Secondary outcomes included postoperative IRP and postprocedural reflux. Treatment failure was defined as the persistence or reoccurrence of an Eckardt score > 3 or the need for retreatment.

**Results:**

A total of 577 patients were included: 498 in the NA group, 15 in the POEM group and 64 in the PD group. Intraoperative mucosal perforation occurred similarly (*p* = 0.65). No mortality was recorded. The median follow-up was 30 months (IQR: 22–34). Treatment success was significantly lower after prior POEM (80%) compared with naïve (98.6%) and PD group (95.3%) (*p* < 0.001). Abnormal postoperative acid exposure was more frequent after prior POEM (33.3%) (*p* < 0.01). In selected patients with recurrence, endoscopic ultrasound revealed marked esophageal wall fibrosis.

**Conclusions:**

LHD is safe and effective even after failed endoscopic therapy. However, patients previously treated with POEM represent a higher-risk subgroup with lower success rates and increased reflux.

Although esophageal achalasia (EA) is one the most common functional disorders of the esophagus, its etiology remains largely unknown [1, 2]. Consequently, current treatment options are limited to symptom palliation rather than disease cure. As a result, many patients may undergo multiple therapeutic interventions over the course of their lifetime [2].

For two decades, pneumatic dilation (PD) and laparoscopic Heller–Dor (LHD) represented the only therapeutic options proven to achieve durable symptom control in patients with EA. LHD has emerged in the last 30 years as the treatment of choice, particularly in young patients [3, 4]. Current clinical guidelines encourage surgical myotomy for patients under 45 years old, given the inferior outcomes associated with PD in this age group, whereas endoscopic approaches are recommended for patients with increased operative risk.

Recently, peroral endoscopic myotomy (POEM) has been introduced into clinical practice and has progressively gained popularity. Being perceived as less invasive and effective, POEM—together with LHD—has become one of the procedures of choice for the treatment of EA. Nevertheless, in a minority of patients (estimated between 10 and 20%), symptoms persist or recur after treatment. The mechanisms underlying treatment failure are often unclear. In some cases, technical factors, such as an incomplete myotomy, may play a role, particularly when failure occurs shortly after the endoscopic procedure or when symptoms persist from the outset. However, in the majority of cases, the underlying cause of treatment failure remains unclear. Consequently, the management of POEM failure is currently based mainly on small case series or case reports [5, 6], underscoring the need for standardized management guidelines.

The aim of this prospective, controlled study was to evaluate the perioperative and functional outcomes of LHD in patients who previously underwent endoscopic treatment—POEM or pneumatic dilatation—as compared to treatment-naïve patients with EA.

## Materials and methods

All consecutive patients with a definitive diagnosis of achalasia who underwent laparoscopic Heller–Dor between 2020 and 2025 at the General Surgery Unit 1, Azienda Ospedale-Università Padova, were enrolled in the study. Patients’ demographic and clinical data, surgical details, and postoperative outcomes were prospectively collected in a dedicated database. In particular, instrumental postoperative data (i.e., endoscopy, esophagogram, manometry, and pH impedance) were collected in all patients who accepted to repeat these tests following the Padua protocol [7]. All procedures were performed according to a standardized surgical technique routinely adopted at our center [8, 9].

Patients were stratified into three groups: treatment-naïve patients (NA group), patients previously treated with an endoscopic myotomy (post-POEM group), and patients who had undergone pneumatic dilation (post-PD group). During the study period, 62 POEM procedures were performed at our institution. However, only two patients included in the present series had undergone POEM at our center, whereas the remaining post-POEM patients were referred from other institutions as a tertiary referral center for salvage treatment. All patients with persistent or recurrent symptoms after previous PD or POEM were discussed at a multidisciplinary meeting. LHD was offered when further endoscopic retreatment was considered less appropriate because of prior treatment failure, unfavorable anatomy, or patient preference.

The primary outcomes were postoperative morbidity, mortality, and therapeutic success. Secondary outcomes included evaluation of postoperative integrated relaxation pressure (IRP) and incidence of postprocedural gastroesophageal reflux. Treatment failure was defined as the persistence or reoccurrence of an Eckardt score > 3 or the need for retreatment [7, 10].

The study was approved by the institutional review board at Azienda Ospedale Università di Padova and by the Research Committee of the Department of Surgical, Oncological, and Gastroenterological Sciences at Padova University.

### Preoperative assessment

The diagnosis of primary achalasia was confirmed by high-resolution manometry (HRM) according to the Chicago Classification v4.0. Symptoms were assessed using the Eckardt Score (ES). Before LHD, all patients underwent barium swallow to assess the diameter of the esophagus [11] and esophagogastroduodenoscopy (EGD) to rule out cardia malignancy, eosinophilic esophagitis, or other esophageal diseases [7].

### Surgical technique

The surgical technique for LHD has been described in detail elsewhere [8, 9]. All procedures were performed by experienced surgeons using a standardized approach. Briefly, only the anterior esophageal wall was dissected and a myotomy 6–8 cm long was performed, extending 1.5–2 cm onto the gastric side. A 30-mm Rigiflex balloon was positioned at the cardia level over an endoscopically placed guidewire and intermittently inflated (40–60 cc of air) to expose circular muscle fibers and facilitate safe division. The edges of the myotomy were separated and peeled away from the submucosal plane. Minimal bleeding from submucosal vessels was easily controlled by inflating the balloon, thus reducing the use of the cautery. The operation was completed with an anterior partial hemifundoplication (180°) according to Dor.

### Postoperative management and follow-up

All patients underwent a water-soluble contrast swallow (Gastrografin®, Bracco, Milan, Italy) on the first postoperative day. If no leak was detected, a liquid diet was started, followed by soft foods starting on postoperative day 2.

The postoperative follow-up included examining patients one, 6, and 12 months after LHD, and every 2 years thereafter, using the ES that was administered preoperatively. Barium swallow X-ray was required one month after LHD and in case of recurrent symptoms. In patients presenting with recurrent symptoms, barium swallow was additionally reviewed to assess for structural postmyotomy abnormalities, including blown-out myotomy (BOM), defined as a distal wide-mouthed (> 2 cm) pseudodiverticular dilation at the level of the previous myotomy associated with a > 50% increase in esophageal diameter compared with the proximal segment.

HRM was performed 6 months after LHD, together with 24-h pH monitoring, to assess esophageal function and acid exposure. Endoscopy was recommended one year after LHD, and then every 2 years thereafter, to identify and control any complications of gastroesophageal reflux and rule out any (albeit rare) malignant degeneration.

In patients with persistent or recurrent symptoms after LHD, further evaluation with endoscopic ultrasound (EUS) was performed, in order to investigate the potential structural causes such as esophageal wall fibrosis or incomplete myotomy.

### Statistical analysis

Continuous variables were reported as medians (IQR). Categorical data were compared using Fisher’s exact test and continuous variables using the Kruskal–Wallis test. No post hoc pairwise comparisons were drawn because they were not of interest to the authors for the purposes of the present study. Pre- to postoperative variations were assessed using Wilcoxon’s signed rank test. A two-sided p-value of less than 0.05 was considered significant. The statistical analyses were performed using SAS 9.1 software.

## Results

A total of 577 patients underwent laparoscopic Heller–Dor myotomy during the study period: 498 in the NA group, 15 in the post-POEM group (two patients had two POEM before the LHD), and 64 in the post-PD group.

### Baseline characteristics

The patients’ demographic and clinical characteristics are summarized in Table 1. Sex distribution was similar across groups, while patients previously treated with PD were significantly older compared with the other groups (*p* = 0.001). Preoperative ES did not differ significantly among groups. Radiological stage distribution differed significantly, with more advanced stages observed in previously treated patients (*p* < 0.001). Esophageal diameter was progressively larger from naïve to post-treatment groups (37 vs 45 vs 50 mm, *p* < 0.001). Manometric parameters also differed significantly: lower preoperative LES resting pressure was observed in previously treated patients (*p* = 0.008), together with lower integrated relaxation pressure (IRP) (p = 0.004).

**Table 1:**
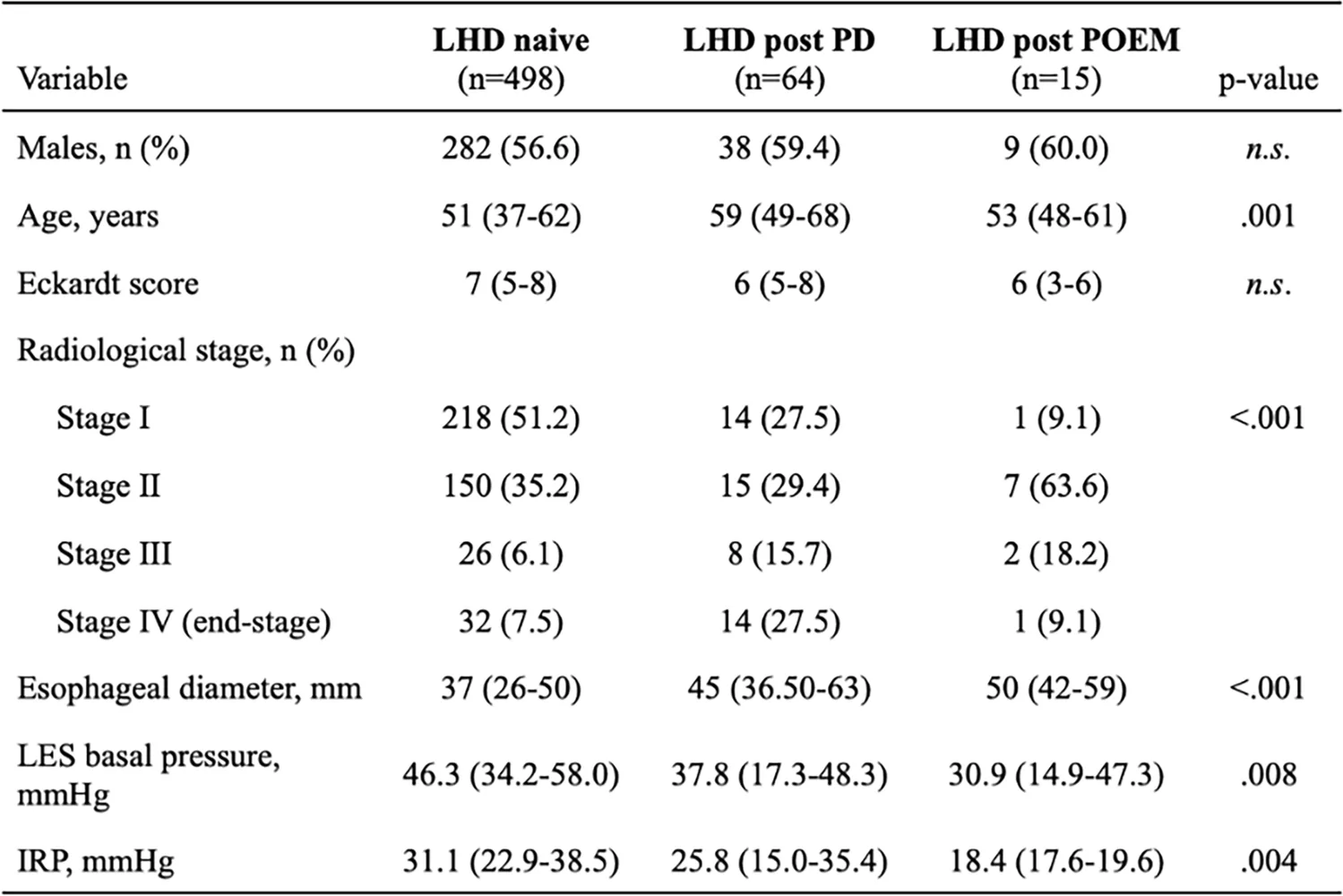
Demographic and preoperative clinical data

Data are summarized as *n* (%) or median (IQR). Percentages are reported according to the number of patients with available data for each variable analyzed. *LHD* laparoscopic Heller-Dor, *PD* pneumatic dilation, *POEM* peroral endoscopic myotomy, *PSEI* inferior esophageal sphincter pressure, *IRP* integrated relaxation pressure, *n.s.* not significant

The median interval between POEM and salvage LHD was 30 (IQR 19–36) months, whereas the median interval between the last preoperative pneumatic dilation and salvage LHD was 24 (IQR 6–84) months.

### Intraoperative and postoperative outcomes

Intraoperative and postoperative outcomes are summarized in Table 2. Operative time and myotomy length were comparable among the three groups. All procedures were completed laparoscopically, with anterior myotomy and hemifundoplication according to Dor. Only in two patients, with previous POEM at the anterior esophageal wall, the redo myotomy was performed on the right esophageal wall to avoid the fibrotic plane and perform the redo myotomy in a naive wall.

**Table 2:**
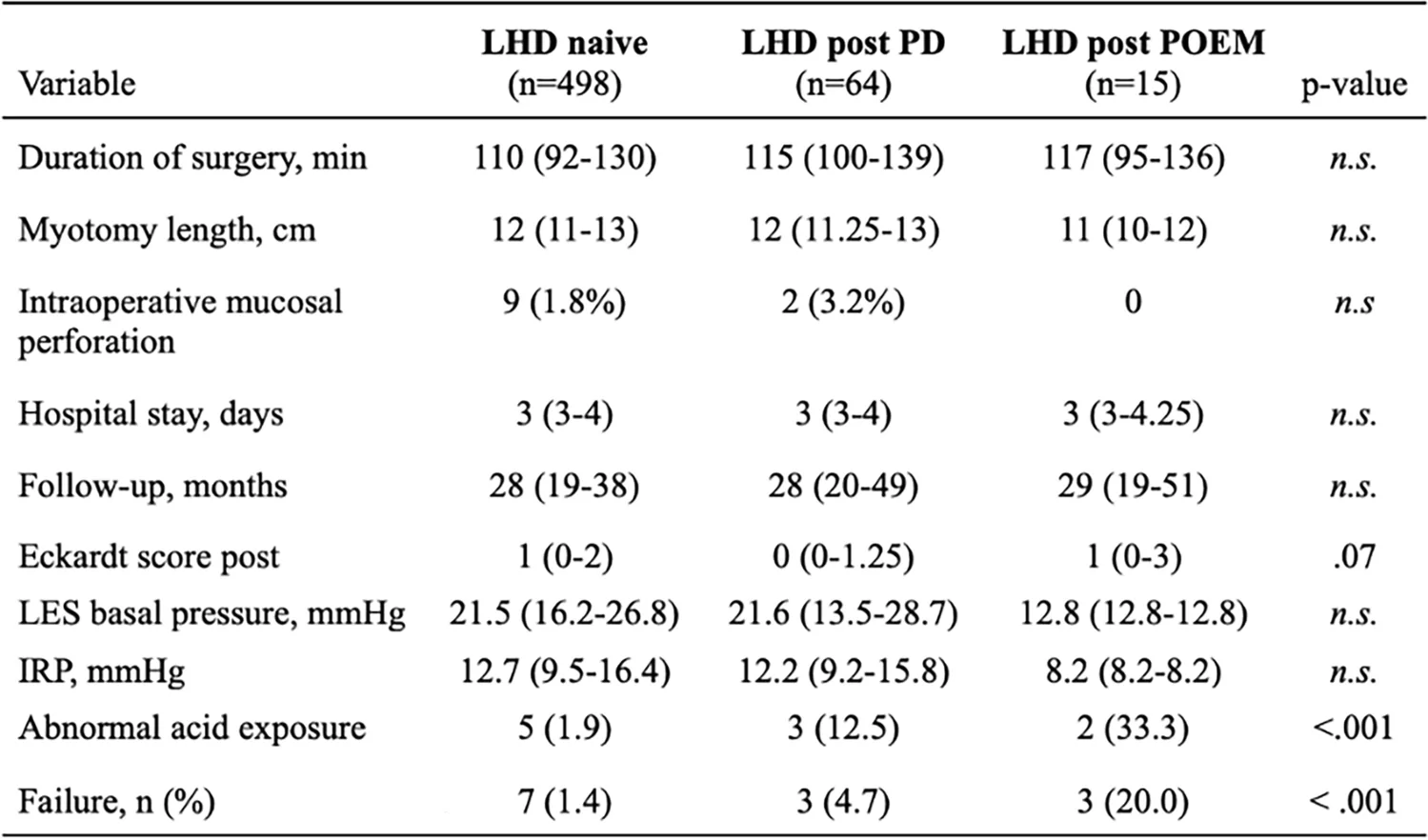
Intra- and postoperative data

Data are summarized as *n* (%) or median (IQR). Percentages are reported according to the number of patients with available data for each variable analyzed. *LHD* laparoscopic Heller-Dor, *PD* pneumatic dilation, *POEM* peroral endoscopic myotomy, *LES* lower esophageal sphincter, *PSEI* inferior esophageal sphincter pressure, *IRP* integrated relaxation pressure, *n.s.* not significant

Intraoperative mucosal perforation rates were similar: 9 (1.8%) patients of the NA group, 2 (3.2%) of the PD group, and no-one in the POEM group (*p* = 0.65). No mortality was recorded. The median follow-up was 30 months (IQR: 22–34).

Postoperative symptom control was good in all groups, with low ES and no statistically significant differences (*p* = 0.07). Similarly, postoperative LES basal pressure and IRP did not differ significantly. However, 24 h pH monitoring after LHD showed a higher incidence of abnormal postoperative acid exposure in the post-POEM group (33.3%) compared to the other two groups: 1.9% in the NA and 12.5% PD groups (*p* < 0.01).

### Treatment failure and evaluation of recurrent symptoms

At a median of follow-up of 30 months (similar between the 3 groups), treatment failure differed significantly, occurring more frequently after previous treatments and reaching the highest rate in post-POEM patients (1.4% vs 4.7% vs 20.0%, *p* < 0.001). Failure of LHD was managed with PD in all cases; symptom persistence was observed in only one patient in the NA group and in all three patients in the post-POEM group. Among patients with persistent symptoms after LHD in the post-POEM group, blown-out myotomy (BOM) was identified on barium swallow in all patients and an esophageal stenosis was detected above the LES.

**Fig. 1:**
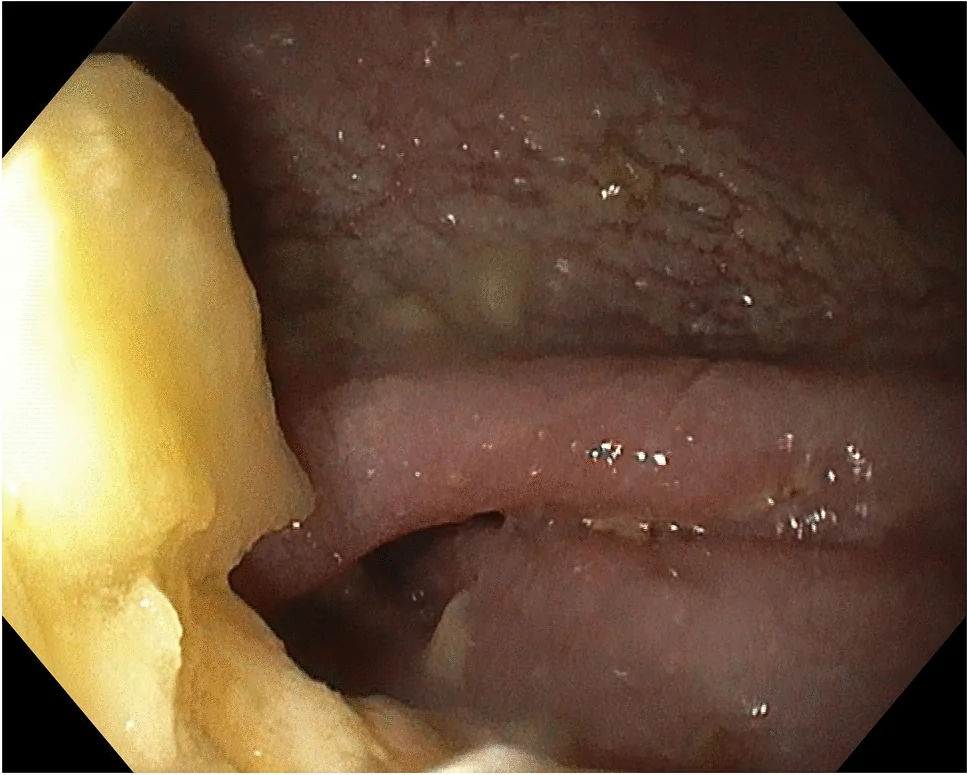
Endoscopic findings in post-POEM patients with recurrent symptoms after laparoscopic Heller–Dor myotomy (LHD)

**Fig. 2:**
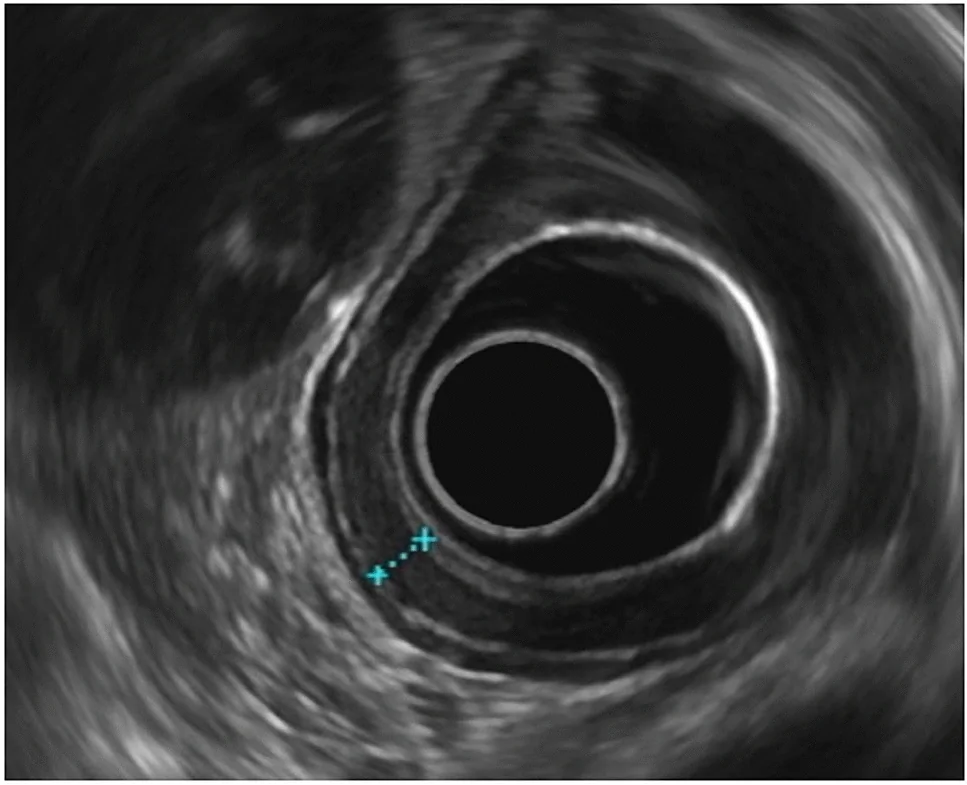
Endoscopic ultrasound (EUS) findings in post-POEM patients with recurrent symptoms after laparoscopic Heller–Dor myotomy (LHD)

Two of the three post-POEM patients who experienced recurrence after LHD underwent additional evaluation with endoscopic ultrasound (EUS) to investigate the possible underlying causes of symptom persistence. EUS demonstrated loss of the normal wall stratification at the myotomy side, with the presence of mildly hypoechoic tissue predominantly involving submucosal layers, compatible with fibrosis. Proximal to the myotomy site, focal (non-circumferential) thickening of the fourth layer (muscularis propria) is noted, compatible with the underlying disease (Fig. 1 and 2).

## Discussion

In this study, we evaluated the outcomes of LHD myotomy in treatment-naïve patients compared with those previously treated with pneumatic dilation (PD) or peroral endoscopic myotomy (POEM). Our findings indicate that prior endoscopic therapy does not adversely affect operative feasibility or perioperative safety. Specifically, previous treatment with PD did not influence the outcomes of LHD, whereas patients with prior POEM showed significantly higher rates of treatment failure.

This issue has been strenuously debated in literature: some authors have reported that prior endoscopic treatments may increase technical difficulty in performing myotomy, complication rate, and/or negatively affect the final outcome [12–15]; whereas others have suggested no such effects of previous treatments [16–19]. In a previous study from our group [20], we observed no influence of previous, failed PD on the outcome of LHD. On the other hand, recently van Hoeij and colleagues [21] showed that, in patients with persistent or recurrent symptoms after POEM, retreatment with LHD achieved a positive outcome in less than half patients. In our study, the success rate was 80%; however, prior POEM still appears to negatively influence the final outcome of LHD.

The mechanisms underlying POEM failure are not yet fully understood. A first explanation could be a structural remodeling and fibrosis at the gastroesophageal junction, potentially complicating surgical dissection. Histopathological studies [22] have demonstrated that achalasia is frequently associated with smooth-muscle fibrosis, eosinophilic infiltration, increased TGF-β1 expression, and collagen deposition, with more severe fibrosis correlating with poorer clinical response after POEM and worse long-term symptom control. Consistent with this hypothesis, in two patients from our cohort with recurrence after LHD, endoscopic ultrasound demonstrated marked esophageal wall thickening and fibrosis, suggesting that advanced submucosal scarring may reduce surgical effectiveness in selected cases. An additional mechanism that may explain the higher retreatment rate observed in the post-POEM group is the development of a blown-out myotomy (BOM). In a large tertiary-center experience [23], BOM was identified in a relevant proportion of symptomatic patients after myotomy and was associated with significantly worse post-treatment Eckardt scores and a higher rate of clinical failure. Consistent with these findings, BOM was detected in 20% of post-POEM patients in our cohort, which is comparable to the 17.8% rate previously reported.

Overall, our findings are consistent with previous reports [24, 25] showing that prior treatment does not necessarily compromise symptomatic palliation after myotomy. In particular, a recent propensity-matched study [23] from Jain et al. reported no statistically significant differences in long-term symptom palliation (ES ≤ 3), IRP, or LES basal pressure between groups, although there is a subtle trend toward slightly increased reintervention rate in patients with prior interventions.

Importantly, prior endoscopic treatment did not significantly affect operative duration or mucosal perforation rates. This suggests that, in experienced hands and with standardized technique, LHD remains technically feasible even after prior interventions. While some studies [26] report increased perforation rates in patients with prior endoscopic interventions, particularly PD, due to altered tissue planes and scarring, other publications [20, 24] have found no statistically significant difference in perforation rates between patients with prior treatment and those who are treatment-naive, suggesting that outcomes may be more influenced by other risk factors, such as advanced age, long disease duration, and esophageal dilation.

It is well established that the risk of gastroesophageal reflux disease (GERD) after POEM ranges between 35 and 50% [27], and some authors suggest performing an additional (in a second step) laparoscopic fundoplication in case of GERD complications [28]. Our study is the first in literature that evaluates the incidence of GERD in patients undergoing POEM and additional LHD in case of recurrence. In our series, we observed a significantly higher incidence of abnormal acid exposure in previously treated patients after POEM. It is reasonable to believe that the structural and functional effects of two myotomies (the endoscopic first and the surgical after) may compromise the efficacy of Dor fundoplication, leading to persistent or recurrent gastroesophageal reflux. Similarly, in our previous experience with redo Heller myotomy, postoperative GERD was significantly more frequent than after primary myotomy despite the routine addition of a fundoplication [9]. This finding supports careful postoperative surveillance in patients undergoing LHD after previous POEM [29], regardless of symptom status given the greater incidence of adenocarcinoma recorded in achalasia-treated patients [30]. Follow-up should include postoperative endoscopy every two years (according to our institutional protocol), together with HRM and 24-h pH monitoring to evaluate the incidence of postoperative reflux and the need of medical therapy. Future studies should investigate whether alternative antireflux strategies may further reduce postoperative GERD in these patients.

Taken together, our results suggest that LHD remains a safe and effective treatment for achalasia regardless of prior therapy. However, patients previously treated, especially after POEM, may represent a higher-risk subgroup with increased postoperative reflux and higher likelihood of retreatment. These findings should be interpreted with caution, as patients undergoing salvage LHD after POEM represent a highly selected subgroup referred for tertiary care after treatment failure. Therefore, the observed differences cannot be attributed solely to the previous endoscopic myotomy.

The strengths of this study include the large single-center cohort, the prospectively maintained database, the standardized surgical technique, and the detailed functional follow-up including HRM and postoperative pH monitoring. Several limitations must be acknowledged. The relatively small number of post-POEM patients limits statistical power for subgroup comparisons and requires cautious interpretation of treatment success and postoperative outcomes. Although this represents one of the largest reported surgical series in the literature, the findings require confirmation in larger multicenter studies. In addition, complete baseline data were not consistently available for patients previously treated with PD or POEM, as many underwent their primary treatment at other institutions before referral to our tertiary center for salvage surgery. Furthermore, the follow-up duration, while adequate for mid-term outcomes, does not allow assessment of long-term outcomes.

## Conclusions

Laparoscopic Heller–Dor appears to be safe and provides effective symptom control in patients with achalasia regardless of previous endoscopic treatment. However, prior interventions, particularly POEM, are associated with increased postoperative reflux and higher failure rates, although outcomes remain good in the mid-term. Further studies are necessary to clarify the mechanism of recurrence in these patients and improve treatment success.

## References

[1] Savarino EV, Salvador R, Ghisa M, Mari A, Forattini F, Costantini A, De Giorgio R, Zaninotto G (2024) Research gap in esophageal achalasia: a narrative review. Dis Esophagus. 10.1093/dote/doae024 38525929

[2] Boeckxstaens GE, Zaninotto G, Richter JE (2014) Achalasia. Lancet 383:83–93. 10.1016/S0140-6736(13)60651-0 23871090

[3] Gockel I, Junginger T, Eckardt VF (2006) Long-term results of conventional myotomy in patients with achalasia: a prospective 20-year analysis. J Gastrointest Surg 10:1400–1408. 10.1016/j.gassur.2006.07.006 17175461

[4] Triadafilopoulos G, Boeckxstaens GE, Gullo R, Patti MG, Pandolfino JE, Kahrilas PJ, Duranceau A, Jamieson G, Zaninotto G (2012) The Kagoshima consensus on esophageal achalasia. Dis Esophagus 25:337–348. 10.1111/j.1442-2050.2011.01207.x 21595779

[5] D’Angelo A, Pioche M, Barret M, Gonzalez J-M, Wallenhorst T, Coron E, Velut G, Vanbiervliet G, Onana Ndong P, Jacques J, Schaefer M, Levy J, Caillo L, Rahmi G, Aidibi A, Ah-Soune P, Degand T, Saunier M, Zerbib F, Berger A (2026) RedoPOEM: efficacy and safety after failure of a first POEM for esophageal motility disorders. Surg Endosc 40:3090–3098. 10.1007/s00464-026-12564-4 41575544

[6] Wessels EM, Masclee GMC, Bastiaansen BAJ, Saleh CMC, Tack J, Boeckxstaens G, Costamagna G, Familiari P, Fockens P, Schijven MP, Bisschops R, Bredenoord AJ (2026) Long-term outcomes of peroral endoscopic myotomy (POEM) versus pneumatic dilation after prior laparoscopic Heller myotomy for achalasia. Clin Gastroenterol Hepatol. 10.1016/j.cgh.2026.03.024 41932531

[7] Costantini M, Salvador R, Capovilla G, Vallese L, Costantini A, Nicoletti L, Briscolini D, Valmasoni M, Merigliano S (2019) A thousand and one laparoscopic Heller myotomies for esophageal achalasia: a 25-year experience at a single tertiary center. J Gastrointest Surg 23:23–35. 10.1007/s11605-018-3956-x 30238248

[8] Ancona E, Peracchia A, Zaninotto G, Rossi M, Bonavina L, Segalin A (1993) Heller laparoscopic cardiomyotomy with antireflux anterior fundoplication (Dor) in the treatment of esophageal achalasia. Surg Endosc 7:459–461. 10.1007/BF00311744 8211631

[9] Capovilla G, Salvador R, Provenzano L, Valmasoni M, Moletta L, Pierobon ES, Merigliano S, Costantini M (2021) Laparoscopic revisional surgery after failed Heller myotomy for esophageal achalasia: long-term outcome at a single tertiary center. J Gastrointest Surg 25:2208–2217. 10.1007/s11605-021-05041-x PMC8484080 34100246

[10] Capuano M, Palomba G, Pesce M, Fernicola A, Efficie E, De Palma GD, Sarnelli G, Aprea G (2026) Clinical utility of Eckardt score and high-resolution manometry in the follow-up of achalasia patients treated with Heller–Dor myotomy. Curr Probl Surg 74:101929. 10.1016/j.cpsurg.2025.101929 41513386

[11] Patti MG, Feo CV, Diener U, Tamburini A, Arcerito M, Safadi B, Way LW (1999) Laparoscopic Heller myotomy relieves dysphagia in achalasia when the esophagus is dilated. Surg Endosc 13:843–847. 10.1007/s004649901117 10449836

[12] Horgan S, Hudda K, Eubanks T, McAllister J, Pellegrini CA (1999) Does botulinum toxin injection make esophagomyotomy a more difficult operation? Surg Endosc 13:576–579. 10.1007/s004649901044 10347294

[13] Patti MG, Feo CV, Arcerito M, De Pinto M, Tamburini A, Diener U, Gantert W, Way LW (1999) Effects of previous treatment on results of laparoscopic Heller myotomy for achalasia. Dig Dis Sci 44:2270–2276. 10.1023/A:1026660921776 10573373

[14] Morino M, Rebecchi F, Festa V, Garrone C (1997) Preoperative pneumatic dilatation represents a risk factor for laparoscopic Heller myotomy. Surg Endosc 11:359–361. 10.1007/s004649900363 9094277

[15] Souma Y, Nakajima K, Taniguchi E, Takahashi T, Kurokawa Y, Yamasaki M, Miyazaki Y, Makino T, Hamada T, Yasuda J, Yumiba T, Ohashi S, Takiguchi S, Mori M, Doki Y (2017) Mucosal perforation during laparoscopic surgery for achalasia: impact of preoperative pneumatic balloon dilation. Surg Endosc 31:1427–1435. 10.1007/s00464-016-5133-1 27501729

[16] Bonavina L, Incarbone R, Reitano M, Antoniazzi L, Peracchia A (2000) Does previous endoscopic treatment affect the outcome of laparoscopic Heller myotomy? Ann Chir 125:45–49 10921184

[17] Ferguson MK, Reeder LB, Olak J (1996) Results of myotomy and partial fundoplication after pneumatic dilation for achalasia. Ann Thorac Surg 62:327–330 8694585

[18] Holzman MD, Sharp KW, Ladipo JK, Eller RF, Holcomb GW, Richards WO (1997) Laparoscopic surgical treatment of achalasia. Am J Surg 173:308–311. 10.1016/S0002-9610(96)00398-4 9136786

[19] Tsuboi K, Omura N, Yano F, Kashiwagi H, Kawasaki N, Suzuki Y, Yanaga K (2009) Preoperative dilatation does not affect the surgical outcome of laparoscopic Heller myotomy and Dor fundoplication for esophageal achalasia. Surg Laparosc Endosc Percutan Tech 19:98–100. 10.1097/SLE.0b013e31819cb127 19390272

[20] Portale G, Costantini M, Rizzetto C, Guirroli E, Ceolin M, Salvador R, Ancona E, Zaninotto G (2005) Long-term outcome of laparoscopic Heller-Dor surgery for esophageal achalasia: possible detrimental role of previous endoscopic treatment. J Gastrointest Surg 9:1332–1339. 10.1016/j.gassur.2005.10.001 16332491

[21] van Hoeij FB, Ponds FA, Werner Y, Sternbach JM, Fockens P, Bastiaansen BA, Smout AJPM, Pandolfino JE, Rösch T, Bredenoord AJ (2018) Management of recurrent symptoms after per-oral endoscopic myotomy in achalasia. Gastrointest Endosc 87:95–101. 10.1016/j.gie.2017.04.036 28478028

[22] Zhao W, Wang B, Zhang L, Zheng Z, Chen X, Zhao C, Wang B, Jin H, Zhang L (2022) Effect of esophageal muscle fibrosis on prognosis of per-oral endoscopic myotomy (POEM) in achalasia patients. Surg Endosc 36:7477–7485. 10.1007/s00464-022-09172-3 35534736

[23] Triggs JR, Krause AJ, Carlson DA, Donnan EN, Campagna RAJ, Jain AS, Kahrilas PJ, Hungness ES, Pandolfino JE (2021) Blown-out myotomy: an adverse event of laparoscopic Heller myotomy and peroral endoscopic myotomy for achalasia. Gastrointest Endosc 93:861-868.e1. 10.1016/j.gie.2020.07.041 PMC7855725 32721488

[24] Jain N, Barron JO, Toth AJ, Sudarshan M, Sanaka M, Ramji S, Adhikari S, Murthy SC, Blackstone EH, Raja S, Cleveland Clinic Esophageal Research Group (2025) Heller myotomy in patients with prior endoscopic interventions vs the treatment-naïve. Surg Endosc 39:3328–3336. 10.1007/s00464-025-11661-0 PMC12041173 40232403

[25] Werner YB, Hakanson B, Martinek J, Repici A, von Rahden BHA, Bredenoord AJ, Bisschops R, Messmann H, Vollberg MC, Noder T, Kersten JF, Mann O, Izbicki J, Pazdro A, Fumagalli U, Rosati R, Germer C-T, Schijven MP, Emmermann A, von Renteln D, Fockens P, Boeckxstaens G, Rösch T (2019) Endoscopic or surgical myotomy in patients with idiopathic achalasia. N Engl J Med 381:2219–2229. 10.1056/NEJMoa1905380 31800987

[26] Smith CD, Stival A, Howell DL, Swafford V (2006) Endoscopic therapy for achalasia before Heller myotomy results in worse outcomes than heller myotomy alone. Ann Surg 243(5):579. 10.1097/01.sla.0000217524.75529.2d PMC1570551 16632991

[27] Abu-Nuwar MR, Eriksson SE, Sarici IS, Zheng P, Hoppo T, Jobe BA, Ayazi S (2023) GERD after peroral endoscopic myotomy: assessment of incidence and predisposing factors. J Am Coll Surg 236:58–70. 10.1097/XCS.0000000000000448 PMC9750114 36519909

[28] Ďuriček M, Demeter M, Bánovčin P (2024) POEM in the esophagus - how to deal with the post-POEM reflux. Best Pract Res Clin Gastroenterol 71:101917. 10.1016/j.bpg.2024.101917 39209415

[29] Katz PO, Dunbar KB, Schnoll-Sussman FH, Greer KB, Yadlapati R, Spechler SJ (2022) ACG clinical guideline for the diagnosis and management of gastroesophageal reflux disease. Am J Gastroenterol 117:27–56. 10.14309/ajg.0000000000001538 PMC8754510 34807007

[30] Leijonmarck W, Santoni G, Holmberg D, Spechler S, von Euler-Chelpin M, Birgisson H, Ness-Jensen E, Kauppila JH, Lagergren J (2026) Achalasia and risk of esophageal squamous cell carcinoma and adenocarcinoma in a multinational study. Gastroenterology. 10.1053/j.gastro.2026.03.024 42019768

